# Treating latent tuberculosis infection (LTBI) with isoniazid and rifapentine (3HP) in an inner-city population with psychosocial barriers to treatment adherence: A qualitative descriptive study

**DOI:** 10.1371/journal.pgph.0000017

**Published:** 2021-12-08

**Authors:** Amber Heyd, Courtney Heffernan, Kate Storey, T. Cameron Wild, Richard Long

**Affiliations:** 1 School of Public Health, University of Alberta, Edmonton, AB, Canada; 2 Faculty of Medicine and Dentistry, University of Alberta, Edmonton, AB, Canada; Keele University, UNITED KINGDOM

## Abstract

In Canada, preventive therapy for latent tuberculosis infection (LTBI) has required multiple doses of medication over an extended period of time. Such regimens are associated with poor adherence and completion rates. A shortened treatment regimen of once weekly isoniazid plus rifapentine for 3 months (3HP), is now available, and holds promise in populations facing challenges to treatment adherence. Although many factors impact treatment adherence, a knowledge gap exists in describing these factors in the context of this regimen. We present findings from a qualitative descriptive study, involving semi-structured interviews with unstably housed or homeless individuals in Edmonton and Fort McMurray, Alberta, Canada who were offered directly-observed preventive therapy (DOPT) with 3HP, and their health care providers. Latent content analysis revealed incomplete understandings of LTBI and about the need for preventive therapy. Clients’ motivation to be healthy, alongside education, health care outreach, relationships developed in the context of DOPT, ease of treatment regimen, incentives, and collaboration were all described as supporting treatment completion. Competing priorities, difficulty in reaching clients, undesirable aspects of the regimen and difficulties obtaining and initiating 3HP were identified as barriers. Perceptions of stigma related to LTBI and TB were described by clients in addition to feelings of shame related to their diagnosis. Our study provides insight into LTBI and indicates that multiple interacting psychosocial factors influence preventive therapy access, uptake, and adherence. Findings from this study of both client and provider perspectives can be used to inform and address inequities among individuals experiencing homelessness, and ultimately contribute to a diminished reservoir of LTBI.

## Introduction

Tuberculosis (TB) is a communicable infectious disease caused by organisms of the Mycobacterium *tuberculosis* (*M*.*tb*) [1]. The pathology of TB disease exists along a spectrum influenced by the complex interplay between bacterium, environment, and host [2–4]. A simplification of this spectrum involves, on one end, latent tuberculosis infection (LTBI). LTBI is characterized by an infection with *M*.*tb* without any clinical, radiological, or microbiological evidence of active disease [3]. On the other end of the spectrum is active disease, most typically characterized by recovery and growth of *M*.*tb* in culture from the affected individual. From a public health perspective, TB is as much a reflection of poor social conditions as it is a biomedical condition. Those who are poorest are at highest risk of both exposure to *M*.*tb*, and progression from latent infection to active disease [5].

Worldwide estimates indicate that 1.7 billion individuals have LTBI, among whom an estimated 5–15% will progress to active disease [6, 7]. In high-income, low TB incidence countries, vulnerability to exposure is influenced by intermediate factors such as disease prevalence, poorly ventilated congregate settings, and malnutrition [8]. In Canada, for example, while the overall incidence of TB disease is low at 4.9 per 100,000, Indigenous peoples report the highest annual incidence of disease and foreign-born persons report, as a group, the highest proportion of cases [9]. Significantly, TB outbreaks are reported in underserved urban populations, and TB patients experiencing homelessness are nearly three times as likely to die within one year of a diagnosis compared to their housed counterparts [3, 4]. Thus, the association between TB and homelessness is substantial [10].

Homelessness is a significant risk factor for exposure to *M*.*tb*, and progression to disease. People who use inner-city shelters or drop-in centres have a much higher prevalence of LTBI in comparison to the general population (30–40% vs 5–10%, respectively) [10]. Moreover, higher rates of comorbid physical and mental health conditions among individuals experiencing homelessness increase the risk of progression from LTBI to active disease [11, 12].

Treatment of LTBI involves the challenge of convincing otherwise healthy persons of the need to treat an infection that may never develop into active disease with prolonged antibiotic therapy that may cause side effects [5]. In Canada, the standard first-line treatment regimen for LTBI has heretofore been nine months of daily isoniazid (INH-9) [13]. This medication has efficacy rates of 69–93% however, toxicity and long treatment duration with frequent dosing negatively affect completion [13]. As a result, considerable attention has been paid to developing and using shorter treatments that are comparably safe and effective. The most attractive of these is a combination of INH and rifapentine taken once weekly for 12 doses (3HP) [14]. The 3HP regimen for adults is 900mg isoniazid (3 x 300mg tablets) taken with 900mg rifapentine (6 x150mg tablets) alongside vitamin B6 for a total of 10 or 11 tablets per week at one time. Current CDC guidelines recommend this regimen be taken after eating to support increased absorption, and that all pills be swallowed in one sitting, within 5–10 minutes [15]. In order to monitor adverse events, as well as to maximize adherence to a treatment that is only 12 doses—wherein each dose represents a high proportion of the whole regimen—the treatment is administered as directly-observed preventive therapy (DOPT) [14, 16].

Homelessness has been shown to impact LTBI treatment outcomes [12, 17, 18]. Among individuals experiencing homelessness in the UK, a high prevalence of LTBI, co-infection with hepatitis B (HBV) and hepatitis C (HCV), as well as poor treatment uptake and engagement in care was found [18]. In separate inner-city populations, completion of screening requirements for TB/LTBI was reported to be poor (53%), and homelessness was identified as the only factor negatively affecting LTBI treatment outcomes [17, 19]. In a study that offered 6–12 months of twice weekly directly-observed INH preventive therapy (DOPT) to homeless men in Seattle with LTBI, 47 (73%) began therapy, while only 23 (49%) completed [20]. In a study that offered 6 months of INH preventive therapy to homeless adults in San Francisco with LTBI, only 36 (31%) completed [21]. In contrast, Nwana et al (2019) found a high rate of treatment success among individuals experiencing homelessness using 3HP (n = 301/393, 76.6%) [22]. In that study, qualitative interviews with health care providers revealed challenges in following up with persons experiencing homelessness related to transiency, mistrust, and alcohol and/or substance use [22]. However, research examining the use of preventive therapy, and more specifically, 3HP, among individuals experiencing homelessness is limited. In particular, research about its use in Canada, is limited given that the regimen is relatively new. Furthermore, research that employs qualitative methods to explore both the client and provider experiences of using 3HP among persons experiencing homeless is, from our review, non-existent. This study aims to fill this knowledge gap by describing perceptions of LTBI and its treatment among people offered 3HP while unstably housed or homeless, among whom the benefits of treatment completion are great, and their health care provider(s) (HCP). Furthermore, we aimed to increase awareness of the use of this regimen within a Canadian context and from the perspective of a seldom-heard population.

## Methods

### Methodological approach

We used a qualitative descriptive method that provides a basic description and summary of a phenomenon from the perspective of those who live it [23]. This methodology is best used when clear descriptions of phenomena are invited and the researcher wishes to know the who, what, and where of events [23, 24]. The resulting outcome is a descriptive summary of the informational contents of the data, organized in a way that best fits the data [23]. In qualitative descriptive studies, latent content analysis is the analysis strategy of choice [23]. As such, we used principles of latent content analysis, including identifying, coding, and categorizing, to guide our analytic process [23, 24].

### Setting

This study was conducted in the Province of Alberta, Canada population 4,384, 968 in 2019 [25]. Recruitment occurred in two urban centres: Edmonton and Fort McMurray between June 2019 to January 2020.

### Ethics

The study was approved by both the University of Alberta Health Research Ethics Board 3 (Pro00087572) and the Northern Alberta Clinical Trials and Research Centre (NACTRC), a joint venture of Alberta Health Services and the University of Alberta (PB85954).

### Sample, recruitment, and consent

Purposeful homogenous sampling was used to recruit participants who could provide rich information on the phenomenon of interest [26]. Participants were eligible for interviews if they were: English-speaking adults (aged >17 years) in Edmonton or Fort McMurray, Alberta who experienced homelessness or who were unstably housed within 12 months of their LTBI diagnosis, and were offered preventive therapy with 3HP, and declined, accepted but did not complete, or completed treatment with 3HP. Interviews were also performed with health care providers (HCP) who administered 3HP to individuals who were unstably housed/homeless.

An overview of the study was presented to staff of the Provincial TB Program, an outreach team (Street Connect), and to care providers at the Centre of Hope shelter in Fort McMurray, prior to recruitment. Through provincial TB services, and independent from this study, individuals were screened for LTBI through a targeted outreach program; in Fort McMurray, individuals accessing the Centre of Hope were offered information and screening for LTBI when visiting their HCP; in Edmonton, contacts of inner-city individuals with active TB disease were screened, and if determined to have LTBI, offered preventive therapy. The recruitment strategy relied on HCP identifying potential participants who met the inclusion criteria. Prior to notification of the interviewer, potential participants were provided with information about the study by their HCP regarding the study and extended an invitation to participate. If, the client agreed at this point, consent to contact was obtained. Following review of the study information sheet, informed consent was obtained prior to the conduct of interviews. To recruit HCP, the Edmonton TB Clinic, the Provincial TB Clinic, and the Street Connect team in Fort McMurray were contacted by email. If HCP expressed interest in participating, a review of the information sheet and completion of the consent process occurred in person, or in some cases, over the phone. Accordingly, informed written consent was obtained from all participants with signatures collected; witnessed consent from clients was completed with their health care provider, consent provided from health care providers in the study was not witnessed. Verbal consent was collected prior to recording of the interview. All participants were made aware that they could revoke consent and withdraw from the study at any time before or during the interview process. All interviewees were offered a $20 gift card to either 7-Eleven or Starbucks as acknowledgment of their time.

### Data collection

Semi-structured interviews were conducted by the research team. Tailored interview guides were developed for both clients and HCP following a review of the literature as well as feedback provided from experienced TB researchers, and the researchers own experience in clinical TB care and working with persons experiencing homelessness. The guides included questions exploring the experience of being diagnosed with LTBI as well as the offer, acceptance, and completion of 3HP treatment. Examples of interview questions for clients included: Can you tell me what you know about TB? Can you describe the difference between latent and active TB? What helped you complete your treatment for LTBI? For HCP, examples of question include: What do you like best about using 3HP in comparison to other regimens in this population? What do you find challenging about using 3HP? What were your perceptions of 3HP prior to the program using it? Although a set of guiding questions was used, they evolved throughout the data collection phase to further explore emerging themes. Review of the data generated through interviews with a TB physician and a qualitative researcher external to the project, occurred following the first set of interviews and after all interviews had been conducted. This debriefing process assisted the researcher in organizing findings and identifying areas of further exploration in subsequent interviews. Interviews were completed in English and conducted in a variety of settings, for the convenience of the participant and safety of the researcher. Four of nine interviews with HCP were conducted over the phone, all interviews with clients were conducted in person. Consent was obtained for the audio-recording of interviews and they lasted on average 30 minutes. Audio files were sent for transcription and then iteratively reviewed to correct inaccuracies. All data were anonymized by removal of any potential identifiers.

### Analysis

Inductive content analysis has been described as the most fitting analytical technique for an exploratory or descriptive qualitative study [24]. The technique is a method for systematically describing the meaning of qualitative data and includes three key features: it reduces data, it is systematic, and it is flexible [27]. Code and coding frameworks are generated from the data themselves and systematically applied during the analysis process. Development of categories to organize similar codes, and generation of themes, leads to a descriptive summary of the informational content of the data, organized in a way that best fits the data. Latent content analysis is distinct from other forms of content analysis in that the role of the researcher is to discover the implied meaning in participant’s experiences, rather than provide a more surface-level interpretation [28]. Consistent with qualitative descriptive methodology, this process allowed us to focus on staying close to the data, while interpreting the meaning of the narrative.

Beginning the process of analysis, the interviewer familiarized herself with the data by reviewing personal reflections and field notes, documenting the overall impression of the interview, and reflecting on what the participant was trying to convey after each interview was completed. Data were organized using ATLAS.ti [29]. The interviewer then listened to recorded interviews, reviewed and cleaned transcripts to maintain confidentiality. Errors that occurred during transcription were corrected. Following these steps, the interviewer began the process of generating open codes for each transcript. Codes were then assembled into categories, which were then reviewed and merged or collapsed to ensure that the categories themselves were distinct from each other. Guided by the development of categories and subcategories, the interviewer formulated a general description of the research topic and findings, creating a descriptive summary of the contents of the data, organized in a way that best reflected the data [19]. Iteration during analysis allowed for the exploration of topics that were being uncovered.

### Rigour

Throughout, this study followed the set of criteria proposed by Lincoln and Guba (1985) and explored by Tracy (2010), to maintain trustworthiness (rigour) [30, 31]. These criteria are credibility, transferability, dependability, and confirmability and informed the verification strategies used [24, 31, 32].

Strategies used to enhance rigour included the researchers’ experience in the field, peer debriefing, member checking, an audit trail and a personal journal. The researcher spent time with the Street Connect team and at local shelters prior to conducting interviews, and has prior experience working with persons experiencing homelessness. Peer debriefing was completed with three external qualitatively trained researchers at different stages throughout the research process, firstly, after initial interviews were completed and secondly, after data collection had ended. Member checking was completed with HCP, who were invited to review established findings once all interviews were completed. Logistical constraints and feasibility were such that member checking was only completed with HCP of the study. An audit trail was used to record a detailed description of the why, when, and how decisions were made throughout the research process. A personal journal was used to foster reflexivity during the research process and included reflections on how the research was unfolding, interactions with the participants during interviews, biases and theoretical assumptions, as well as challenges and highlights.

## Results

Nineteen interviews were completed; ten with clients and nine with HCP. The mean age of clients was 47 years, and 60% identified as male (Table 1). All clients reported a history of unstable housing or homelessness within a year of their LTBI diagnosis: seven were provisionally housed at the time of the interview, while three were experiencing current homelessness. At the time of interview, six clients completed treatment, one discontinued treatment, and three clients either declined, or delayed treatment. Treatment completion was defined as witnessed ingestion of 11 or 12 doses within 16 weeks of treatment initiation. Delayed treatment refers to an agreement to therapy without having immediately initiated treatment. Seven of the ten interviewees reported previous or current alcohol and/or substance misuse.

**Table 1:** Demographic characteristics of clients (n = 10).

Characteristics	*n (%)*
Sex	
Female	4 (40)
Male	6 (60)
Age, years	
All clients, mean (SD) (range 17–64)	47 (14)
Population Group	
Canadian-born non-Indigenous	4 (40)
Canadian-born Indigenous	3 (30)
Foreign-born	3 (30)
Housing Status	
Low-income housing	5 (50)
Housing first program	2 (20)
Homeless (emergency shelter use, couch surfing or sleeping rough)	3 (30)
Reported previous or current alcohol and/or substance misuse	7 (70)

The nine interviewed HCP held a range of positions that included physicians, a nurse practitioner, registered nurses, a social worker, and an Indigenous cultural helper employed by Alberta Health Services (AHS). All reported experience working with clients and patients experiencing homelessness. All had direct involvement, and experience in providing preventive therapy with 3HP to the study population.

The major findings of this study are presented in two sections. This first section reports knowledge and understandings of LTBI, being diagnosed and the use of preventive therapy from the perspective of the client; provider perspectives were judged to be minimally contributory to this category and were not pursued. The second section reports on the identified barriers and facilitators to treatment uptake and completion from both client and HCP perspectives.

### Knowledge and understandings of LTBI, being diagnosed and the use of preventive therapy

Knowledge of TB, LTBI and the difference between active disease and latency varied among clients. Some clients described TB as a germ, virus, bacteria or disease that affects the lungs, while others reported that they had heard of it, but did not know much. When asked to describe the differences between active TB disease and LTBI, some clients had difficulty in describing what having LTBI meant, while others understood a latent infection to be “dormant” and referred to the terms “sleeping TB” at risk of “waking up.” As one client shares:

Well active… active you’ll get really sick… if you just got the latent germs in your lungs right and they’re dormant… like they can be dormant for years… then all of sudden start up. (P3, client [completed])

The conversation with clients about a diagnosis of LTBI uncovered their experiences of being diagnosed and understanding of their exposures to TB. When asked to share their experience of being diagnosed and how this made them feel, many clients described initial feelings of shock and worry. Many were concerned that they could potentially die from TB disease. As one client indicated:

I’m totally worried. I was, like, “big guy, where else would you take me?” He had me a few times. I’ve been hit by vans and trucks and none of those stopped me. (P8, client [delayed])

Others went on to share how they felt a sense of reassurance after learning the infection was latent, what this meant and how it could be treated before becoming active:

You tested positive… and what the hell… and it was a shock to me… what the hell is going on here…went and met with the doctor… ohhhh it’s TB… but it’s dormant. She explained that it was dormant. Okay… do this pill program. Okay… I’m not going to miss nothin. (P3, client [completed])

During this conversation many clients organically shifted to describing their understanding of different exposures that may have led to their diagnosis. Many identified a causal relationship between being around someone who is coughing and exposure to TB:

Cuz TB like goes around right? They gotta be coughing around you in order for you to catch the germs in your lungs. (P1, client [declined])

Clients also described the misperception that transmission may have occurred through the sharing of personal items, such as drinks, cigarettes and drug equipment. Some expressed a sense of personal responsibly, where infection was associated with “being a bad apple” (P6, client [completed]), while others felt that they were blamed for acquiring the infection. Many clients relayed their belief that experiences associated with homelessness, such as shelter use, sharing items, or being around people who are generally unwell exposed them to TB. Exposure and subsequent infection were connected to alcohol and substance use and being involved in street life. As two clients describe:

I was quite surprised because I knew they said this [diagnosed with LTBI] but I didn’t know who had it. I shared a bottle and cigarette. You can’t finish a cigarette outside the door anyway without sharing when you’re on the streets. Probably everybody’s just passing it on to people or whatever, don’t realize they have it. Being homeless on the streets, lots of that is going on. (P13, client [completed])

I’m a heavy smoker and I kind of led a real hardcore life. I’m not going to lie. I’m on the fucking streets. Partied, did a lot of drugs and shared pipes and shit like that so I probably might have it myself right? Probably one of the reasons why I fucking got it. My addiction led to me getting tuberculosis. I know it was because of the addiction, sharing pipes, smokes, pots, picking buts, sharing smokes and drinks, bottles and whatever else. I don’t use needles, I smoke. [laughs] Maybe the occasional snort. The party life led me to have TB and I want to get rid of it. (P12, client [delayed])

Most clients described how treatment eradicates TB bacteria and shared the perception that preventive treatment provides a means to both reduce risk of active disease and protect others from exposure to infection. As one client shares:

Interviewer: Can you tell me a little bit about why you said yes to taking treatment?

Participant: I don’t know…I just feel like maybe I’m worried that if it got active I can pass it on, I just don’t want to pass it on and give it to somebody else, right? (P8, client [delayed])

During the interview process many clients conveyed a desire to know more about TB. All asked questions about TB and latent infection and were eager to learn. Most reported that they received information about TB from their HCP, however, a few reported that they learned about it from either a friend or family member. Although some clients reported they were informed they had latent infection, as mentioned above, it was still unclear as to what this meant, and they sought clarification of their diagnosis during the interview process. As one client shared:

Do I have TB… I want to see the doctor you know? I want to know 100% whether or not I have TB? (P4, client [discontinued])

Other clients suggested that more information should be made available about TB and LTBI to increase awareness of both, the differences between them, and understanding of the need for treatment.

### Barriers and facilitators to treatment access, uptake, and adherence

**Fig 1:**
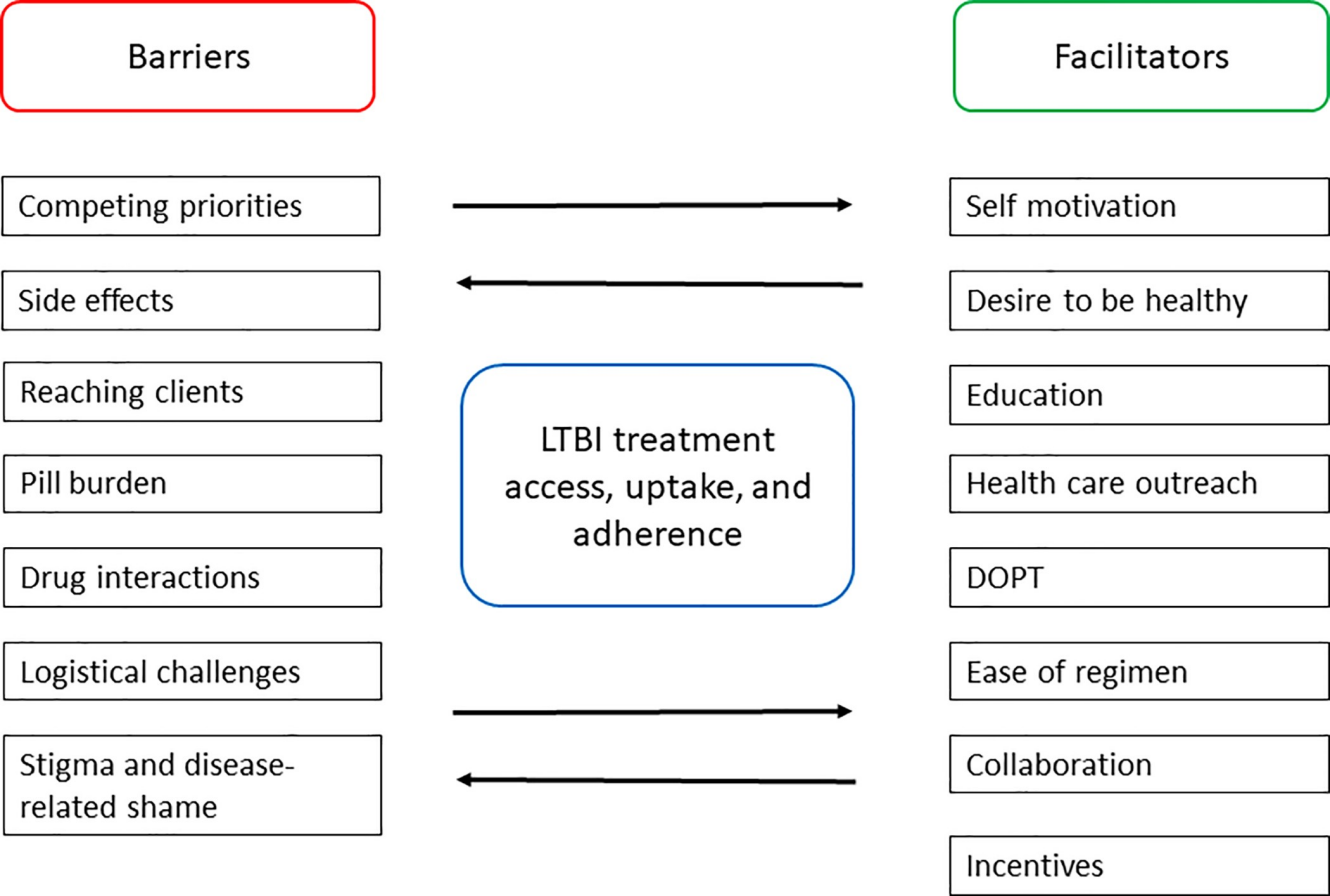
Barriers and facilitators to treatment access, uptake, and adherence.

Interviews with clients and health care providers identified several interacting barriers and facilitators to treatment access, uptake, and completion. A total of seven barriers and eight facilitators were identified. These categories of barriers and facilitators and emerging themes are summarized in Fig 1.

#### Barriers

*Competing priorities*. Instability related to homelessness and competing priorities were challenges to treatment uptake. Clients and HCP discussed how preventive therapy was not a priority and described how this results in delays or non-acceptance of treatment. All participants remarked on how addiction and substance use inhibited accessing care, testing, and treatment for LTBI. In addition to addiction and substance use competing priorities related to unstable housing, unemployment and overall lack of a routine were also described barriers to starting treatment for LTBI. Participants noted how they felt the need to address these basic needs first before beginning treatment:

I was trying to find money or work. Kind of saving. Just trying to stay clean. It’s hard just to keep your sobriety when you’re around everyone that’s fucking doing it. It’s hard. It’s not like going to your house and cooking the meal and watching TV and go to bed. I got to live with that right now, buddy’s helping me out with a place to stay, I’m helping him out, so kind of worked out a deal. Now I’ve got this housing, I’m making steps to get this TB—but you got to get on the schedule and I got to get a place and then so have somewhere to go and I can get myself into that routine and then I can absolutely get on these medicines. Get rid of this TB. Get back my health. So I can get back to what I want to do. Right? (P12, client [delayed])

*Side effects*. A few clients who had initiated treatment reported intolerable side effects from taking 3HP. One in particular reported feeling abdominal pain, dizziness, and nausea while on treatment. This client stopped her treatment after one month and switched to an alternate regimen before she began a one-month religious fast (P4, client [discontinued]). Other clients reported similar concerns related to GI upset or discomfort, as well as some generalized joint/body aches. HCP all reported assessing clients for side-effects at each weekly dose in addition to monitoring routine bloodwork to assess for abnormalities. Most described the regimen as one that is well-tolerated. In line with the description of side-effects clients offered, however, a few HCP indicated that nausea and abdominal discomfort were the most frequently reported complaints while completing the regimen.

*Reaching clients*. Almost all HCP reported that connecting with clients each week was a significant barrier to treatment completion. HCP described how, due to the transient nature of their clients’ lives, they experienced difficulties in reaching the clients in order to dispense 3HP doses weekly. HCP described the effort and flexibility required to find the clients each week, and reported that this took up an appreciable amount of their time. Locations for DOPT ranged from shelters, personal residences, public health centres, to meeting people on the street or local hangout spots. As one HCP mentioned:

The challenges would be just to connect with people. There’s no stable address that we can always find them. Most of our clients don’t have cell phones. Contacting them to try to arrange administering the medication is also a challenge. We spend a lot of time calling clients, old phone numbers, or their friends’ phone numbers. Just try to connect. Then sometimes we might run into the client and not have the medication. Then we’re running around to make it the most efficient as possible and also trying to not make it a huge inconvenience to the client. (P7, HCP [Indigenous cultural helper])

*Pill burden*. The number of pills taken at each dose, in one sitting, was identified as a barrier to treatment initiation and completion by clients and HCP. Many HCP reported concerns regarding pill burden, stating “I knew the pill burden would be a big hurdle to overcome” (P18, HCP [registered nurse]) and described surprised or hesitant reactions of clients when they were made aware of number of pills required to take at each dose. The number of pills were also described as a deterrent to treatment acceptance and uptake:

The biggest thing that limited people wanting to take or ability to finish the 3HP regimen, was the amount of pills. So, a lot of people, at least I can think of two people that didn’t want to take it because there were just too many pills. (P19, HCP [physician]).

*Drug interactions*. A few HCP made remarks related to drug-drug interactions, reporting that clients in the inner-city are often on multiple medications. Concerns with interactions between medications such as mood stabilizers and opioid agonist therapies were reported. Because of the relative novelty and limited use of 3HP in Canada, physicians and nurses recalled not offering the regimen to clients because of these concerns.

*Logistical challenges*. Logistical challenges related to accessing 3HP and performing the pre-treatment investigations necessary before its prescription, were barriers to its uptake. Pre-treatment investigations included those necessary for the exclusion of active TB, such as sputum collection and chest x-ray, and those necessary to the confirmation of LTBI and the baseline stability of hematologic, renal and liver function. As one HCP (P9 [nurse practitioner]) explains “from a health systems approach, the availability of the lab, testing, and X-ray, are barriers to care.” Supply challenges in Fort McMurray, including delays in receiving interferon gamma release assay (QuantiFERON®-TB Gold Plus) collection tubes and the medication itself, as well as the support clients required for completing pre-treatment tasks contributed to a delayed treatment start. HCP noted how the more steps required and the longer treatment start is delayed, the more the risk of losing clients, or clients being unable to complete the regimen.

*Stigma and disease-related shame*. Perceptions of stigma related to LTBI and TB were described by clients as well as feelings of disease-related shame related to their diagnosis. When asked whether or not clients shared their diagnosis with others, most reported that they kept it private and that TB was not discussed among their peers. As one participant shared:

No, because the way I see it here, if anybody got anything, they don’t tell nobody, because that way, they are not embarrassed themselves—or where do you get it? That’s the way it is here. I know of somebody else who was taking the same thing I was, but we never sat down and talked about it or anything. I haven’t seen her for a while, but I know she’s around. She was pretty well going in the same time I was in. I knew, without saying a word. (P6, Client [completed])`

In relation to stigma, some clients described feelings of shame connected with their diagnosis of LTBI. One described a sense of guilt when told of his diagnosis while another became emotional as he described how his relationship had changed with his partner after he shared his diagnosis (P3, client, completed).

Although most participants described satisfaction with DOPT, described in more detail below, a couple of participants spoke to the need for a more “private” space in order to maintain confidentiality and reduce experiences of stigma.

#### Facilitators

*Self-determined motivation*. When clients were asked to share what factors made treatment easier to complete, many went on to describe how they were self-motivated to complete preventive therapy. Clients relayed how the choice to accept treatment was made by themselves. This helped to contribute to a sense of determination to complete therapy despite competing priorities.

Clients also shared how they felt a sense of accomplishment and pride once they finished their treatment. A few HCP also made comments regarding the self-motivation of their clients as a determining factor for treatment completion. As one HCP described:

The ones that continued to the end were the most focused on preventing TB before we even got started. You can tell when people are motivated and saying, "Yes, I want to do this." "You know there’s a lot of pills," and they go, "That’s okay. I want to do this and I want to get it over as soon as possible." The duration of treatment was important to people and they weren’t easily swayed from their target by possible side effects. The ones that were the most motivated to prevent TB in the future were the ones that were successful. (P18, HCP [registered nurse])

*Desire to be healthy*. Clients conveyed that taking treatment for LTBI fit within the bigger picture of a desire to achieve and maintain health. They often described how they were ready to “get back to being healthy” (P12, client [delayed]) and how taking treatment was a part of that process. One participant described how taking treatment is “good for you and good for your health” (P2, client [completed]) while another conveyed “I’m getting up in age, so I want everything checked… everything. I want to try to get as healthy or know what’s wrong before I hit 65” (P6, client [completed]). Participants described using the treatment regimen as an opportunity to put themselves and their health first.

*Education*. HCP highlighted that education about LTBI and preventive therapy facilitates treatment acceptance, and completion. Providers reported that clients were receptive to education and more likely to accept preventive therapy if they understood latency, the need for treatment and the difference between latent and active disease. As one HCP shared:

I think they [nurses] did a really good job of just helping the clients understand what the medications were for. Helping them understand the difference between latent and active I think is a big one for getting a lot of the clients on board because their immediate reaction is like, "ah I have this disease," so just that education piece is important. (P10, HCP [social worker])

When discussing education, most HCP reported that one on one education during interactions and using plain language were the most effective strategies. They described how paper resources such as information sheets are often lost by clients experiencing homelessness.

*Health care outreach*. Programs that provide mobile outreach health care at shelters and drop-in centres accessed by participants facilitated LTBI testing, and treatment uptake and completion. Clients noted how they first learned about TB screening through outreach services either at a drop-in centre, or mobile outreach. Some shared their experiences in accessing care through the shelter or at sites they frequently visited, and how these services supported adherence and completion of their treatment:

I would take the medication in the afternoon…I go for lunch at the Salvation Army church. So then I see the nurses there and say, "Oh, are you coming in this afternoon?" Then I know it’s open and I can take my medication. (P14, client [completed])

Likewise, HCP commented that outreach and meeting clients “where they are at” helps to facilitate treatment completion. As previously described, HCP reported great diversity in the locations and environments where weekly treatments are provided, and noted the importance of services being accessible. As one indicated:

We would try to organize the clients to always administer it on Tuesdays because we’re at the shelter then. If we didn’t see them, we would go to places we knew they stayed at and see if they’re around to take it. We’d sometimes just carry it [3HP dose] in our vehicle so that we would always have it in case we ran into them, to give it to them within the closest time frame that we could. All over town, which is the nature of our team anyways, so it makes sense for us. (P7, HCP [Indigenous cultural helper])

*DOPT*. Clients reported positive experiences of DOPT, which helped with treatment adherence and completion. Clients reported that the continuity of receiving their treatment by weekly DOPT provided an opportunity to develop more meaningful relationships with their providers, ensured that treatment was completed and administered correctly, and overall improved their treatment experience. As one client described:

Interviewer: What was your relationship like with the outreach nurse?

Participant: Well, she’s a friend afterwards. She started to give me the medication over and over and over and again and again and again. We became friends, and that’s more important to me than anything else. (P5, client [completed])

Many clients shared their appreciation of the caring attitudes of the health care team and described how this encouraged them to complete their treatment. When asked if they would have preferred to take treatment on their own, as self-administered therapy (SAT), almost all clients expressed that it was unlikely they would take it on their own and preferred the support provided by receiving the treatment through direct observation. HCP reported that DOPT is critical for ensuring that treatment was completed in whole among those who have risk factors for treatment non-completion and for early detection of adverse events. Both clients and providers noted how the instability in their lives make it challenging to complete on their own. Both HCP and clients relayed how receiving DOPT weekly also provided a means to connect and follow up on other needs as well, such as housing, employment or other health-related concerns. As one HCP shared:

It was nice to have a motivation to see them and they have a reason to see us. It’s a link and we might be there to administer the medication, but you end up providing supports for housing or bringing warm clothes or something like that. I definitely don’t think that the challenges are a reason to stop doing it, but I do think that there needs to be teams like this one or community support services in place. (P7, HCP [Indigenous cultural helper])

*Ease of 3HP regimen*. Both HCP and clients appreciated the once weekly dosing, and the shortened duration of the regimen relative to other options for preventive therapy. Most clients tolerated the regime well when asked to describe their treatment experience. Clients reported that taking treatment was “easy” and “just like anything else” (P6, client [completed]) and appreciated that you complete the regimen once and then it’s done. When asked if they were offered conventional therapy (e.g. 4 months of rifampin or 6–9 months of daily isoniazid self-administered) instead of 3HP, some clients reported that it was unlikely they would be able to complete those regimens on their own.

HCP also reported their preference for the 3HP regimen in comparison to treatment regimens with more frequent dosing and longer durations. They shared how the once per week dosing was more manageable. In inner-city Fort McMurray, one provider shared how she felt that the regimen facilitated treatment completion in individuals that likely would not have succeeded on conventional treatment:

I’d say that this 3HP regimen is very attractive for anyone that would—it’s the regimen itself is fast. It’s not pills every day. It’s pills once a week. We’ve successfully completed five, we have a couple that are waiting. It’s a small population and I do think that those little successes—It doesn’t seem like lots of numbers, but that’s five more people than we ever would’ve gotten on treatment before. I guarantee it. (P11, HCP [registered nurse])

*Collaboration*. HCP expressed the importance of collaboration both within the team, and with outside organizations as key to supporting treatment access, uptake, and adherence among clients. Different team members offered unique supports to clients, in collaboration with other agencies such as pharmacies, additional shelters and treatment centres and health services. As one provider shared:

The healthcare resources were our team. We used the recovery workers or we worked a lot with the nurses. We had to use the lab for doing routine bloodwork. We had to use the diagnostic imaging when we were doing our first screening to make sure that it’s not active. The medication which specifically came from Edmonton and the TB nurse herself. Then the physician is involved so quite a lot of resources for one client but collaboratively ends up with good results. I think that that just shows, that’s what you need for a good result. (P9, HCP [nurse practitioner])

*Incentives*. Providers reported that the use of incentives supported treatment adherence and completion. Although there were no formal incentives used in either Fort McMurray or Edmonton, HCP reported that often informal incentives, such as coffee, bus tickets, a small meal or transportation often motivated clients to meet for their weekly dose.

## Discussion

This study sought to describe perceptions of LTBI and the barriers and facilitators of preventive therapy access, uptake and adherence, using a shortened regimen, (3HP), among individuals experiencing homelessness and their HCP. This is the first study to qualitatively explore the use 3HP from both a client and provider perspective and adds to a very limited literature on these topics. By using qualitative methods, we captured the expressive and meaningful voices of people who are not often represented in TB research, but who experience an increased risk of developing active disease.

The findings revealed that knowledge of LTBI varied among individuals offered preventive therapy. Interviews suggested only a moderate understanding of the difference between active TB disease and latent infection, and even poorer understanding of transmission risks. Clients requested additional information about LTBI during the interview process and raised questions regarding the difference between active TB and latent infection, transmission, treatment and prognosis. These results are consistent with a limited understanding of LTBI identified in other studies. They suggest that education can support treatment uptake and adherence [5, 32–36]. In the current study, clients identified how receiving education on LTBI from their HCP improved their understanding of their diagnosis and the value of preventive therapy while others indicated that an increased awareness of the seriousness of LTBI as it pertains to TB disease, and the role of preventive therapy in dampening that transition could improve uptake and adherence.

In a study that examined multi-level barriers to LTBI treatment among Latino adolescents, Hill et al. (2010) highlighted that many participants required numerous contact attempts for prompting and assistance to complete pre-treatment tasks, suggesting an increased risk of loss to follow up at each stage of the LTBI cascade of care [35]. This was true in our study as well, with HCP remarking on how pre-treatment requirements, including lab work, sputum collection and chest x-ray, delayed treatment uptake and reduced the chance of treatment initiation. In studies exploring LTBI treatment adherence among individuals experiencing homelessness, competing priorities related to meeting basic needs and/or addiction and substance misuse can hinder adherence and completion [22, 37]. In this study, many clients stated that competing priorities related to finding housing, seeking employment or managing addiction and substance misuse were reasons for declining or delaying 3HP uptake and/or treatment interruptions.

With respect to the specific 3HP regimen, HCP in a mixed-methods study from the United States identified similar programmatic and follow up challenges encountered as well as patient difficulty with the pill burden of the regimen [22]. Although clients and HCP reported on the positive aspects of DOPT, described in more detail below, the requirement of 3HP to be directly-observed can be interpreted as a policy barrier to treatment initiation and completion. Receiving LTBI treatment via DOT can be viewed as unacceptable to clients who are completing therapy as a preventative measure. Moreover, relative to self-administration, DOPT is excessively costly to TB programs [38]. In our study, HCP commented on the difficulties they encountered in reaching clients for their weekly dosing, remarking on the number of resources and time required to do so. Requiring 3HP to be administered DOPT has been described in the literature as a barrier to using it, and more recent work in jurisdictions with more experience administering 3HP have reported similar completion rates when 3HP is self-administered [16, 38]. However, it is important to note that these studies are centered on populations who may require less psychosocial support for treatment completion than individuals who are unstably housed or homeless. Moreover, while recent changes to the Centers for Disease Control (CDC) guidelines allow 3HP to be administered by either DOPT or SAT in the USA, this is not yet so in Canada. Currently, in Canada rifapentine is only available through Health Canada’s Urgent Public Health Need pathway and it must be administered by DOPT [39]. This directive was purposeful and aimed at allowing Canadian providers to familiarize themselves with the regimen and confirm its safety profile in 3HP-naive populations, many of whom live remote [39]. Administration by DOPT provides opportunity for further population specific evaluations on the safety profile of the regimen, as well as may optimize adherence to a treatment with only 12 doses [13, 14, 16]

With regard to facilitators, our study revealed multiple factors that contribute to improved preventive therapy access, uptake, and adherence using a shortened treatment regimen, 3HP. Clients self-motivation and desire to be healthy, alongside education, health care outreach, relationships through DOPT, ease of treatment regimen, incentives and collaboration were described as supporting successful treatment outcomes. These results are aligned with existing literature on strategies to improve adherence to LTBI preventive therapy. These have demonstrated the effects of incentives on increasing completion rates among LTBI clients with psychosocial barriers to treatment adherence, including a study by Tulsky et al. (2000) which demonstrated an 18% increase in completion rates among adults who were homeless in the United States and a study by Chaisson et al. (2001) which found that among persons who use drugs (PWUD), those who received immediate incentives had higher LTBI preventive therapy completion rates than those who received deferred incentives (83% vs 75%) [21, 40]. As in the literature, our own findings indicate that educational outreach and nurse-led case management, including healthcare outreach, psychosocial support and linkages to medical and social services were found to support treatment uptake and adherence [33, 34, 36, 41, 42]. DOPT was identified as a way to coordinate the provision of these services in our study. Many clients referred to developing relationships with their care provider through the duration of their treatment at their weekly visit for observed dosing. HCP reported the same, stating that the weekly DOPT appointment promoted relationship building and provided an opportunity to address other health and social issues in addition to ensuring the treatment was completed. The shorter treatment duration and less frequent dosing of the 3HP regimen were identified as facilitators to treatment uptake and adherence in our study by both clients and HCP. Clients reported that they would be unlikely to complete both a longer daily regimen and conceptually preferred DOPT over SAT.

Feelings of stigma, shame and embarrassment related to TB in general, and the diagnosis of LTBI specifically, were described by clients. In other literature examining beliefs and attitudes towards LTBI, studies have shown similar experiences of stigma. In a study by Joseph et al. (2004) examining health care workers adherence to occupational TB screening and treatment, stigma was associated with TB disease and infection, with participants noting that they were treated differently by co-workers who may not have understood the difference between LTBI and TB disease [34]. We identified comparable findings in our study, with most clients reporting how they kept their diagnosis and treatment for LTBI private. Although this theme was uncovered during client interviews, it was not revealed in interviews with HCP. This suggests that the stigma described by clients is perceived, reflecting their perception of how others may act or feels towards them, if aware of their LTBI diagnosis [43]. This differs from enacted stigma, which describes discriminatory acts or behaviours [43]. Furthermore, perceived associations of TB with poverty, malnutrition, lifestyle choices and substance abuse, as well as negative stereotypes of individuals experiencing homelessness may contribute to the heightened sense of stigma expressed in our study population [34, 44]. Further research from an intersectional lens or syndemics framework is needed to further our understanding of how perceived stigma impacts TB screening rates among priority populations, as well as its effect on preventive therapy uptake and adherence.

### Implications for practice

The results of this study may inform the development of interventions and health policy to promote LTBI preventive therapy uptake and completion among underserved populations.

As knowledge has been shown to be a facilitator of adherence, targeted education on LTBI and TB may improve LTBI screening and preventive therapy uptake and completion among individuals experiencing homelessness [36]. As stigma and shame related to TB are often fuelled by misperceptions of transmission, disease severity, and a lack of understanding the difference between latency and active disease, increasing educational outreach may be a way to diminish the social stigma surrounding TB [33, 45, 46]. In addition, HCP and TB programs should also be cognizant of locations for DOPT to promote confidentiality and minimize perceptions of feelings of stigma among individuals receiving treatment. Furthermore, peer support and the development of “TB clubs” which meet, or are facilitated online, have been shown to offer social and treatment adherence support empowering individuals with TB and shifting broader community norms [44].

Our study also highlights the potential of more integrated models or approaches to care. In Fort McMurray, care was provided to clients in the study through the multidisciplinary Street Connect program. Integrating biomedical or communicable disease systems care with social services, and ensuring services are accessible and reach target populations, while reducing the silos in primary health care for individuals with complex health and social needs can support TB care and adherence to treatment. This is an area of programmatic research which requires further exploration.

Lowering the pill burden by using a simpler fixed-dose combination (e.g. three tablets combined rifapentine 300mg and isoniazid 300mg) may improve treatment acceptance and adherence rates [47]. Furthermore, streamlining of pre-treatment requirements to reduce delays to treatment initiation may also improve uptake and adherence. As identified in our study, clients who were self-motivated were likely to succeed with treatment completion. As such, including motivational interviewing, adherence coaching and self-esteem counselling as well as peer-based interventions may also improve completion rates [48].

Finally, and arguably most importantly, is that interventions to support preventive therapy access, uptake, and completion do not occur in isolation from attention to and improvement of social determinants of health that confound TB elimination efforts. As a symptom of social inequity, TB requires socioeconomic interventions, including addressing the root causes of homelessness [49]. At scale, national poverty reduction has been shown to reduce TB incidence and reductions in TB incidence have been made through improvements in socioeconomic conditions [47, 50, 51]. While our study identified ways to support and facilitate preventive therapy access, uptake and completion, we do not intend to draw attention away from the social conditions that render persons vulnerable to exposure to M.tb and progression to disease.

### Limitations

This study has some limitations. Firstly, the sample size was limited due to a variety of factors. A limited number of individuals were offered and either declined, discontinued, or completed 3HP during the study time frame. In addition, the transient nature of the population and competing priorities created challenges for client recruitment. As well, due to logistical constraints, member checking was only completed with HCP and the researcher was not able to review findings with the clients to ensure accurate interpretation of their interview data.

### Conclusion

Individuals experiencing homelessness who have LTBI are less likely to complete preventive therapy, but greatly benefit from its use. This study is the first in Canada to qualitatively explore the use of 3HP and adds to the small but growing body of literature on LTBI and use of this regimen. Our study provides insight into the knowledge and understandings of LTBI and the multiple psychosocial factors which interact to influence preventive therapy access, uptake, and adherence. Removal of the LTBI reservoir, referred to as the “seedbeds” of TB disease, is key to elimination and a critical component of the World Health Organization’s End TB strategy [52]. Findings from our study can be used to inform health policy and TB programming aimed at removal of this reservoir and to address TB inequities among individuals experiencing homelessness.

## Supporting information

S1 File Qualitative interview guide. (DOCX)
