# Supplementary material for: Treating latent tuberculosis infection (LTBI) with isoniazid and rifapentine (3HP) in an inner-city population with psychosocial barriers to treatment adherence: A qualitative descriptive study
Source: PLOS Glob Public Health. 2021 Dec 8;1(12):e0000017. doi: 10.1371/journal.pgph.0000017 (PMC10021900; doi:10.1371/journal.pgph.0000017)
Supplement: S1 File — (DOCX) [file pgph.0000017.s001.docx]

# **Interview Guide (Client)**

Group 1: Offered, Accepted and Completed Treatment

1. Can you tell me about yourself? How long have you been in Edmonton (or Fort McMurray)?
2. Can you tell me what you know about TB?
3. Could you describe to me what latent or sleeping TB means to you?
4. Can you tell me what you know about the difference between latent TB and active TB?
5. Can you tell me about when you were first tested for LTBI?
6. Can you tell me about when you were first told that you had LTBI?
   1. What was that experience like?
   2. How did you feel?
   3. What did you say?
   4. What did you do?
7. When you were first diagnosed with LTBI and told that you were exposed to TB did you share this with anyone?
8. Is latent TB or TB something that you talk about with your friends or family?
9. Could you tell me if or how your diagnosis of LTBI affected your relationships?
10. Can you tell me about LTBI and your daily life?
    1. Does LTBI impact you? If so, how?
11. Can you tell me what you know about treatment for LTBI?
    1. Is this something that is important to you?
12. Have you ever been offered LTBI treatment before, not including this most recent time?
    1. Can you tell me a bit more about this experience? Did you accept or decline treatment?
13. What influenced your decision on whether to accept or decline treatment this time?
14. Could you tell me about the medication that you took most recently for treating your latent TB?
15. Could you describe to me what your treatment experience has been like?
    1. What made your treatment easier?
    2. What made your treatment difficult?
16. Can you tell me about your experience with directly observed treatment, where someone gives your medication to you?
    1. Is this something you felt comfortable with?
    2. Can you tell me about who provided your DOT? What was your relationship like with this person?
17. Could you tell me about any of the benefits that you received from completing treatment for latent TB?
    1. How and/or why are these benefits important?
18. Could you describe to me any negative effects that you experienced while completing treatment?
19. Is there anything that you would suggest to that would improve treatment completion?

These next questions focus on your living situation and experience of homelessness:

1. Could you tell me about your current living situation?
   1. Could you tell me about how long you have experienced homelessness or unstable living conditions?
2. Could you describe to me if or how homelessness or your current housing situation affected your decision to accept treatment for latent TB?
3. Thinking about the last few months, are there anyways that your housing situation or homelessness has impacted your ability to complete the treatment?

Group Two: Offered, Accepted, did not complete treatment

1. Can you tell me about yourself? How long have you been in Edmonton (or Fort McMurray)?
2. Can you tell me what you know about TB?
3. Could you describe to me what latent or sleeping TB means to you?
4. Can you tell me what you know about the difference between latent TB and active TB?
5. Can you tell me about when you were first tested for LTBI?
6. Can you tell me about when you were first told that you had LTBI?
   1. What was that experience like?
   2. How did you feel?
   3. What did you say?
   4. What did you do?
7. When you were first diagnosed with LTBI and told that you were exposed to TB did you share this with anyone?
8. Is latent TB or TB something that you talk about with your friends or family?
9. Could you tell me if or how your diagnosis of LTBI affected your relationships?
10. Can you tell me about LTBI and your daily life?
    1. Does LTBI impact you? If so, how?
11. Can you tell me what you know about treatment for LTBI?
    1. Is this something that is important to you?
12. Have you ever been offered LTBI treatment in the past, not including this most recent time?
    1. Can you tell me a bit more about this experience? Did you accept or decline treatment?
13. What influenced your decision on whether to accept or decline treatment this time?
14. Could you tell me about the medication that you took for treating your latent TB?
15. Could you describe to me what your treatment experience has been like?
    1. What made your treatment easier?
    2. What made your treatment difficult?
16. Can you tell me about your experience with directly observed therapy, where someone gives you your medication?
    1. Is this something you felt comfortable with?
    2. Can you tell me about how provided your DOT? What was your relationship like with this person?
17. Could you tell me about any of the benefits that you received from completing treatment for latent TB?
18. Could you describe to me any negative effects that you experienced while completing treatment?
19. Can you describe to me the factors that led to your decision to discontinue the treatment?
20. Is there anything that you would suggest to that would improve treatment completion?

These next questions focus on your living situation and experience of homelessness:

1. Could you tell me about your current living situation?
   1. Could you tell me about how long you have experienced homelessness or unstable living conditions?
2. Could you describe to me if or how homelessness or your current housing situation affected your decision to accept treatment for latent TB?
3. Thinking about the last few months, are there anyways that your housing situation or homelessness has impacted your ability to complete the treatment?

Group 3: Offered, declined treatment

1. Can you tell me about yourself? How long have you been in Edmonton (or Fort McMurray)?
2. Can you tell me what you know about TB?
3. Could you describe to me what latent or sleeping TB means to you?
4. Can you tell me what you know about the difference between latent TB and active TB?
5. Can you tell me about when you were first tested for LTBI?
6. Can you tell me about when you were first told that you had LTBI?
   1. What was that experience like?
   2. How did you feel?
   3. What did you say?
   4. What did you do?
7. When you were first diagnosed with LTBI and told that you were exposed to TB did you share this with anyone?
8. Is latent TB or TB something that you talk about with your friends or family?
9. Could you tell me if or how your diagnosis of LTBI affected your relationships?
10. Can you tell me about LTBI and your daily life?
    1. Does LTBI impact you? If so, how?
11. Can you tell me what you know about treatment for LTBI?
    1. Is this something that is important to you?
12. Have you ever been offered LTBI treatment in the past, not including this most recent time?
    1. Can you tell me a bit more about this experience? Did you accept or decline treatment?
13. What influenced your decision on whether to accept or decline treatment this time?
14. Can you tell me if you would be interested in the future of taking treatment for latent TB?
    1. What are some of the reasons or factors that change your decision to accept treatment in the future?

These next questions focus on your living situation and experience of homelessness:

1. Could you tell me about your current living situation?
   1. Could you tell me about how long you have experienced homelessness or unstable living conditions?
2. Could you describe to me if or how homelessness or your current housing situation affected your decision to accept treatment for latent TB?
3. Is there anything that you would suggest to that would improve treatment acceptance and completion rates among individuals like yourself, who may be experiencing homelessness to treatment?

# **Interview Guide (HCP)**

Health Care Provider Interview Guide

1. Can you tell me about yourself? How long have you been working in the inner-city (or as part of the TB outreach team)?
2. When did your program start using 3HP for LTBI treatment?
3. Was the use of 3HP targeted at a specific population, if so, which ones?
4. Did your program initially plan on treating individuals who are homeless or unstably housed?
5. What were the required resources to provide 3HP to individuals who are unstably housed or homeless?
6. Were than any additional cost implications of using 3HP among this population?
7. What resources did you find helpful in communicating effectively with patients about taking 3HP?
8. Are there any other resources or tools that you think would be useful for health care providers using 3HP in this population?
9. What are your eligibility criteria for treating a patient for LTBI with 3HP?
10. Can you describe the overall process of targeting LTBI screening and 3HP implementation?
11. What were the perceptions and expectations of using 3HP before your program started using it?
12. Was treatment directly-observed?
    1. If yes, can you describe the setting where this occurred? E.g. clinical setting, pharmacy, home
13. How were side effects monitored and addressed? Was this done any differently than how it is for other self-administered LTBI treatment regimens?
14. What do you find challenging about using 3HP in persons experiencing homelessness?
15. What were the experiences patients expressed about 3HP?
16. Would you say there is a difference in using 3HP among those experiencing homeless in comparison to individuals who are stably housed or institutionally housed?
17. What do you like best about using 3HP in comparison to other treatment regimens for LTBI among persons experiencing homelessness?
18. Are there any challenges you faced while using 3HP among this population?
    1. Are there any additional challenges you think other health care providers or facilities might face in adopting the use of 3HP?
19. Overall, based on your experiences, do you feel that 3HP should or should not be used in patients who are unstably housed or experiencing homelessness?
